# Individual Variation in Levels of Haptoglobin-Related Protein in Children from Gabon

**DOI:** 10.1371/journal.pone.0049816

**Published:** 2012-11-20

**Authors:** Heather J. Imrie, Freya J. I. Fowkes, Florence Migot-Nabias, Adrian J. F. Luty, Philippe Deloron, Stephen L. Hajduk, Karen P. Day

**Affiliations:** 1 Peter Medawar Building for Pathogen Research and Department of Zoology, University of Oxford, Oxford, United Kingdom; 2 Department of Microbiology, Division of Parasitology, New York University School of Medicine, New York, New York, United States of America; 3 Institut de Recherche pour le Développement, UMR 216 Mère et Enfant Face aux Infections Tropicales, Paris, France; 4 Faculté de Pharmacie, Université Paris Descartes, Sorbonne Paris Cité, France; 5 Department of Biochemistry and Molecular Biology, University of Georgia, Athens, Georgia, United States of America; London School of Hygiene and Tropical Medicine, United Kingdom

## Abstract

**Background:**

Haptoglobin related protein (Hpr) is a key component of trypanosome lytic factors (TLF), a subset of high-density lipoproteins (HDL) that form the first line of human defence against African trypanosomes. Hpr, like haptoglobin (Hp) can bind to hemoglobin (Hb) and it is the Hpr-Hb complexes which bind to these parasites allowing uptake of TLF. This unique form of innate immunity is primate-specific. To date, there have been no population studies of plasma levels of Hpr, particularly in relation to hemolysis and a high prevalence of ahaptoglobinemia as found in malaria endemic areas.

**Methods and Principal Findings:**

We developed a specific enzyme-linked immunosorbent assay to measure levels of plasma Hpr in Gabonese children sampled during a period of seasonal malaria transmission when acute phase responses (APR), malaria infection and associated hemolysis were prevalent. Median Hpr concentration was 0.28 mg/ml (range 0.03–1.1). This was 5-fold higher than that found in Caucasian children (0.049 mg/ml, range 0.002–0.26) with no evidence of an APR. A general linear model was used to investigate associations between Hpr levels, host polymorphisms, parasitological factors and the acute phase proteins, Hp, C-reactive protein (CRP) and albumin. Levels of Hpr were associated with Hp genotype, decreased with age and were higher in females. Hpr concentration was strongly correlated with that of Hp, but not CRP.

**Conclusions/Significance:**

Individual variation in Hpr levels was related to Hp level, Hp genotype, demographics, malaria status and the APR. The strong correlations between plasma levels of Hp and Hpr suggest that they are regulated by similar mechanisms. These population-based observations indicate that a more dynamic view of the relative roles of Hpr and Hpr-Hb complexes needs to be considered in understanding innate immunity to African trypanosomes and possibly other pathogens including the newly discovered *Plasmodium* spp of humans and primates.

## Introduction

Haptoglobin-related protein (Hpr) is found within trypanosome lytic factor (TLF), the component of human plasma (and that of some old world primates) which kills *T. b. brucei* [1]. Thus *T. b. brucei* is not infective for humans, although it causes a chronic wasting disease, nagana, in cattle [1], [2]. TLF is present in plasma in two forms. TLF-1 occurs within the high-density lipoprotein (HDL) fraction of plasma and, like other HDL molecules, its structure is complex, comprising a cholesterol ester core and an outer layer of phospholipid, neutral lipid and the integrated lipoproteins, Hpr and apolipoprotein L-I (apoL-I) [3]. A proportion of Hpr and apoL-I, together with apoA-1 and IgM is also present in lipid-poor complexes, namely TLF-2 [4].

Hpr is found predominantly as a 45 kD heterodimer consisting of one α- and one β-chain covalently bound together to form (αβ)-dimers [5]. A small proportion is found as an (α2β2)-tetramer [5]. The sequence of Hpr is >90% identical with that of the acute phase protein haptoglobin (Hp) [5]. Hp is comprised of α- and β-chains forming tetramers (phenotype Hp1-1) and polymers (phenotypes Hp2-1 and Hp2-2) [6]. The Hp α-chain is encoded by two co-dominant alleles, *Hp^1^* (of which there are two suballeles; *Hp^1S^* and *Hp^1F^*), and *Hp^2^* [7], [8]. The α^2^-chain is almost twice as long as the α^1^-chain, having evolved via a duplication event [8]. Phenotypic variation between Hp 1F and Hp1S, where F indicates fast and S slow migration during electrophoresis, is dependent upon minor amino acid differences [8].The primary function of Hp is to bind hemoglobin (Hb), the β-chain of Hp binding to one Hb (αβ) subunit [9]. This enables the safe removal of Hb via CD163-mediated internalisation in macrophages and results in rapid clearance of Hp [5], [10]. *T. b. brucei* also takes up Hp-Hb complexes, but via an unrelated surface receptor (TbHpHbR), which facilitates endocytosis and lysosomal localization, the heme moiety being liberated and incorporated into hemoproteins [11]. The Hb-binding region is conserved within Hpr facilitating high affinity Hb binding (K*d*>10^−9^ M) [5], [11]. The *T. b. brucei* surface receptor binds Hpr-Hb complexes within TLF, thus internalizing the toxic HDL leading to lysosomal membrane disruption and parasite lysis [12], [13]. In contrast to Hp-Hb, Hpr-Hb does not bind to mammalian CD163 and the mechanism whereby Hpr-Hb complexes are cleared from plasma, in the absence of trypanosomes, is unknown [5].

Hpr and Hp are primarily expressed in the liver, however, Hpr transcript levels were determined to be only 6% of those of Hp due to the presence of a retroviral-like element in intron 1 [14]. Levels of Hpr have been found to be 10-fold lower than those of Hp (0.03–0.04 mg/ml and 0.27–1.39 mg/ml respectively) [15], [16]. Hp is an acute phase protein i.e. one whose concentration changes by at least 25% during inflammatory disorders [17]. During an acute phase response (APR), interleukin-6 (IL-6) results in up-regulation of Hp production, levels increasing 2-4-fold [17]. The *Hp* and *Hpr* genes are only 2.2 kbp apart and have similar promoter regions [18]. IL-6 responsive elements (IL-6RE) have been identified in both the Hp and Hpr promoters [19]. Whether Hpr levels change in response to IL-6, particularly during an APR, is yet to be elucidated.

Hpr levels have not been studied at a population level and the association between Hpr, Hp and the APR is unknown, particularly in relation to malaria-induced hemolysis. Consequently, we developed a specific ELISA that distinguishes between Hp and Hpr. Hpr levels were measured, together with Hp and the acute phase proteins, C-reactive protein (CRP) and albumin, in a large cohort of children living in a malaria-endemic area of Gabon. We investigated the association of Hpr levels in relation to these acute phase proteins as well as Hp genotype, demographics and malariometric indices. As a comparison, levels of Hpr were also measured in healthy children from the United Kingdom who do not experience such a chronic burden of infectious disease and were not exposed to malaria.

## Methods

### Study Population

Details of the Gabon study design, population and laboratory methods have been published previously [20], [21]. Briefly, the study was undertaken in two villages (Dienga and Bakoumba) in South-East Gabon near the Congo border. Malaria is highly endemic with peaks of transmission at the end of the rainy seasons from September-December and in March-June. [22]. The samples were collected May-June when malaria transmission is intense. There are no tsetse flies in Dienga and Bakoumba, but trypanosomiasis is present in cattle and man in other areas of Gabon [23], [24]. A cross-sectional survey was conducted in May 2000 in a cohort of 741 asymptomatic children aged 1–12 years. Plasma samples were available for determination of Hpr levels in 553 children.

### Ethics Statement

Ethical clearance was obtained from the “Comité d’Ethique Pour La Recherche En Médecine Humaine” as well as the Ministry of Public Health and the Governor of the Province. After consultation with the villages’ authorities regarding an acceptable procedure, a village-wide information meeting was held. In Bakoumba, a public convocation was addressed to all inhabitants where those present included medical authorities (Director of the Hospital, Head of the medical laboratory), local authorities (Sous-Préfet, school headmaster, chief of SODEPAL: Société D’Exploitation du Parc de la Lékédi and traditional leaders) as well as leaders of the project at CIRMF (Centre International de Recherches Médicales de Franceville). The research project was presented orally and discussed. Additional information was provided through the director of SODEPAL (Jean Bourgeais) and the medical staff of the hospital. In Dienga, information was provided orally by the staff of the health center (a nurse and a laboratory worker recruited among villagers). People understood that samples would be used for research on malaria, in relation to red blood cell polymorphisms. The verbal consent of parents/guardians was sought and was given, after allowing time for appropriate consultation, via the representatives of parent associations. Parents that were unwilling for their children to participate were identified and their children subsequently excluded, without prejudice, from study surveys. The Comité d’Ethique Pour La Recherche En Médecine Humaine was informed that an individual verbal consent was obtained and agreed to this, literacy levels being low. Ethical approval was also granted by the Institutional Review Board of the New York University School of Medicine.

Samples taken from 3–4 year old Caucasian children allowed comparison of Hpr levels in two different populations. These serum samples were obtained from stored serum previously collected from healthy children as part of studies conducted by the Oxford Vaccine Group. The use of such samples for further studies was approved by the Oxford Clinical Research Ethics Committee as C02.013 ‘Use of stored serum samples held by the Oxford Vaccine Group to further vaccine related research’.

### Demographic, Parasitological and Genetic Variables

Age and sex were recorded for each child. Parasite densities of *Plasmodium* species were counted on Giemsa stained thick blood smears, and were recorded as the number of parasites/µl of blood, assuming an average leucocyte count of 8000/µl [25]. Hp genotype (including 1F and 1S subtypes) was determined by PCR, using the method of Yano *et al* (1998) with modifications [21], [26].

### Plasma Protein Levels

#### Haptoglobin-related protein

Hpr was detected using a capture enzyme-linked immunosorbent assay (ELISA) which was developed using a specific anti-Hpr monoclonal antibody and polyclonal anti-Hp which cross-reacts with Hpr [27]. The monoclonal antibody, SF14.11, was prepared by hyper-immunization of mice with purified TLF-1 and was identified by screening hybridoma culture for neutralization of TLF killing of *T. b. brucei* [28]. This antibody is specific for Hpr and reacts with native human Hpr in immunoprecipitation and non-reducing western blot assays. Reactivity of the antibody with Hpr is abolished by reduction with either dithiothreitol or β-mercaptoethanol. On western blots the SF14.11 reacts predominantly with the 45 kDa Hpr heterodimer consisting of one α- and one β-chain and weakly with the 92 kDa Hpr heterotetramer containing two α- and two β-chains [27]. It was found to be necessary to purify the antibody from ascites fluid by passing over a protein G column, otherwise at all concentrations used, very slow development and low optical density (OD) values were obtained with all ELISA methods attempted.

Initially various concentrations of polyclonal anti-Hp were used as capture antibody, followed by varying concentrations of pooled plasma (containing Hp and Hpr) (neat to 1 in 1,000,000 dilutions in 3%BSA/PBS) or purified Hp1-1 (Sigma) (100 mg/ml to 0.1 ng/ml), then anti-Hpr (diluted to 10 µg/ml PBS) and finally anti-mouse-conjugate. If polyclonal anti-Hp was used as capture antibody, the ELISA was non-specific, as cross-reaction with Hp occurred. Attempts to remove Hp by means of Hb bound to 1 µm aliphatic amine latex beads (Interfacial Dynamics Corporation, USA) were unsuccessful, only removing just over half of Hp. Alternative methods were attempted using anti-apolipoprotein A-I as capture antibody and anti-Hpr as detector antibody and vice versa but no significant OD values were obtained.

The method which was found to be sensitive and relatively specific was to use the monoclonal anti-Hpr as capture antibody and the polyclonal anti-Hp as detection antibody. The anti-Hpr antibody was diluted in PBS (10 µg/ml) and 100 µl aliquots used to coat 96-well plates (Nunc, Hereford, UK) overnight at 4°C. Following washes (PBS/0.05% Tween 20) and blocking (3% BSA in PBS), the samples, diluted 1∶1000 in 3%BSA/PBS, were added and the plate incubated at room temperature for 60 minutes. Detection was by rabbit anti-human Hp (Sigma, Poole, UK), diluted to 3.33 µg/mL, followed by goat anti-rabbit IgG alkaline phosphatase conjugate (Sigma, Poole, UK), diluted to 46 ng/mL. The substrate was p-nitrophenyl phosphate (Sigma, Poole, UK) at 1 mg/ml in 10% diethanolamine, containing 0.5 mM MgCl_2_, pH 9.8. The reaction was allowed to proceed for 1 hour and the OD at 405 nm was measured. The ELISA was calibrated using purified Hpr as a standard prepared by detergent solubilization of human HDL followed by affinity chromatography using a matrix of anti-Hpr (SF14.11) coupled to Affi-gel 10 beads (Bio-Rad) [3]. The total protein in the preparation was measured by the Lowry method and the proportion corresponding to Hpr was determined by densitometry; the component proteins in the preparation were separated on SDS-PAGE gel, stained with Coomassie blue and the proportion within each band was measured using a Fluor-S densitometer (Bio-Rad, Hemel Hempstead, UK). It was found that 56% by weight of the total protein corresponded to the Hpr αβ-dimer and 24% to the tetramer, the remainder being albumin. Thus, 80% of the total protein was Hpr, which corresponded to 218 µg/ml. Further dilutions of this preparation were used to make standards for the ELISA.

The reproducibility of the ELISA was investigated by taking 10 plasma samples from random UK blood-donors (courtesy of John Radcliff Infirmary) and running the samples on the same and different ELISA plates. The intra-plate coefficient of variation (CV) was found to be 4.2%. The inter-plate CV was 21%, but for the majority of samples the CV was <15% with only a few samples with low Hpr concentrations giving higher CV values.

**Figure 1:**
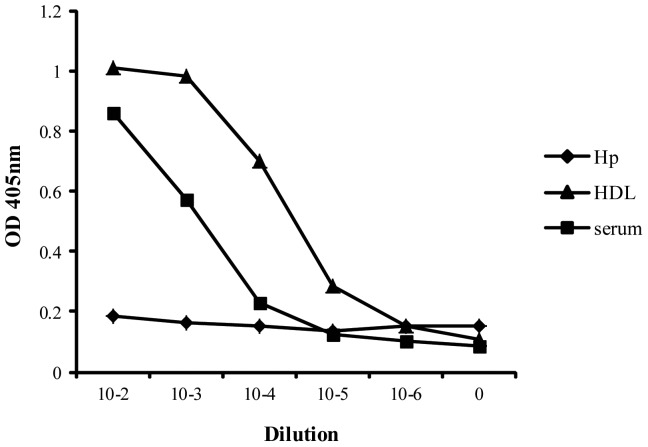
Specificity of Haptoglobin-related protein (Hpr) ELISA. To investigate whether the Hpr ELISA was specific for Hpr rather than Hp, it was performed using various dilutions pooled Hp (Sigma) (stock concentration = 100 µg/ml). To determine whether the ELISA detected Hpr in HDL, it was performed using various dilutions of a purified HDL fraction (stock concentration = 22.5 mg/ml total protein of which 300 µg/ml was found to be Hpr as determined using the Hpr standard). To show that the ELISA could detect Hpr in plasma, it was performed with dilutions of pooled sera (Courtesy of John Radcliffe blood bank). All results are means of duplicate readings and are minus readings from blank wells.

The similarity in structure between Hpr and Hp might have caused cross-reactivity within the ELISA, particularly since it has previously been shown that 0.9% of plasma Hp associates with HDL [29]. This will be a constant factor that will be detected by the reporter anti-Hp polyclonal antibody. Nevertheless, an ELISA that used monoclonal anti-Hpr as capture antibody and rabbit polyclonal anti-Hp as detector antibody was specific for capturing Hpr only. This assay could detect a protein in plasma and in a purified HDL fraction but not in a preparation of purified Hp1-1 (at protein levels ≤1 µg/ml) (Figure 1). Also, addition of pooled Hp up to 4 mg/ml to plasma samples did not affect results.

#### Haptoglobin

Plasma Hp levels were determined by ELISA [21]; 96-well plates were coated with rabbit anti-human Hp (H-8636, Sigma) as capture antibody. Samples were diluted 1 in 10,000 in 3% BSA/PBS and Hp standards (100 ng/ml, 80 ng/ml, 60 ng/ml, 40 ng/ml, 20 ng/ml and 0 ng/ml) prepared using pooled Hp (Sigma) in 3% BSA/PBS. Detection was by monoclonal anti-Hp (Sigma; H-6395) followed by sheep anti-mouse IgG alkaline phosphatase conjugate (Sigma; A3563). Substrate (p-nitrophenyl phosphate) was added and OD read at 405 nm. A standard curve was drawn to determine levels of Hp [21]. Hypohaptoglobinemia is defined as levels <0.18 mg/ml [30].

#### C-reactive protein

Levels of CRP were determined using a commercial ELISA kit (American Laboratory Products Company, Windham, NH, USA). The cut off value to define an APR is ≥10 µg/ml [17].

#### Albumin

Albumin concentrations were determined by mixing 280 µl bromocresol green reagent (Abbott Laboratories Ltd, UK) and 2.4 µl sample in duplicate and reading optical density at 630 nm. Blanks (water), standards (25, 57 g/l) and a quality control (29 g/l) were also prepared. The mean blank value was subtracted from all mean values. A graph was drawn using the two calibration points (straight line up to 60 g/l) and sample values read off. Hypoalbuminemia is defined as levels <35 mg/ml [31].

### Statistical Analysis

Differences between categorical variables were assessed using chi-square tests. Differences between categorical variables and continuous variables were assessed using Mann Whitney *U* or Kruskal-Wallis for non-normal data and t-tests for normal data. Non-parametric analyses between continuous variables were assessed by Spearman correlations.

A general linear model was used to examine the effect of variables of interest on Hpr levels in Gabon. Since Hpr, Hp and CRP levels showed heteroscedasticity, the levels were log transformed before analysis. Variables of interest included CRP concentration (continuous and 2 categories), Hp (continuous and 2 categories), albumin (continuous and 2 categories), *Plasmodium* (log_10_(Plasmodium+1) and 0/1), Hp genotype (3 and 6 categories), age (continuous) and sex. Variables significantly associated with Hpr in the univariate analysis were added to a multivariate model and variables with *P*>0.05 removed in a stepwise fashion. 539 children contributed to the final model (missing values, n = 14). SPSS for Windows version 12.0 (SPSS, Inc., Chicago, IL) was used for data analysis.

## Results

### Hpr Levels in Gabonese and Caucasian Children

**Figure 2:**
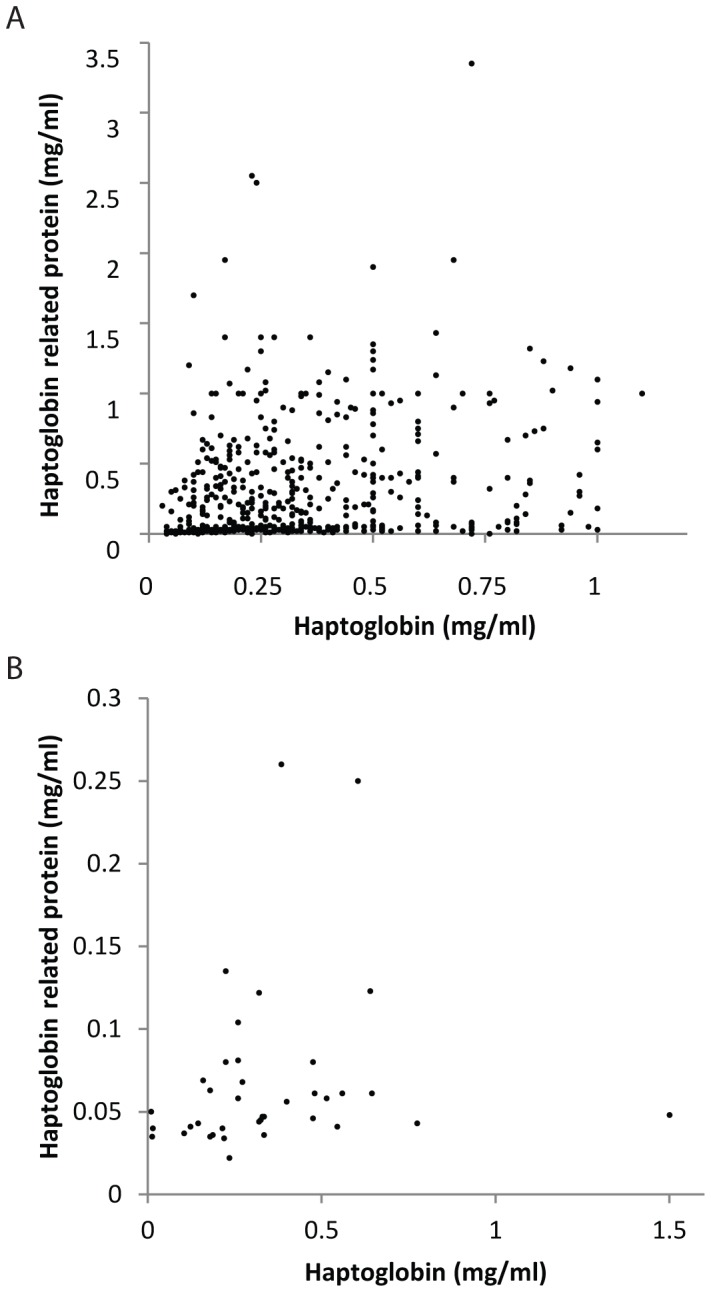
Haptoglobin related protein levels are correlated with haptoglobin levels in A) Gabonese and B) Caucasian children. Scatterplot of raw Hpr values (mg/ml) plotted against raw Hp values (mg/ml). Associations were assessed by Spearmans rho. Hpr was positively correlated with Hp in A) Gabonese children (r_s_ = 0.29, *P*<0.001), and B) Caucasian children (r_s_ = 0.51, *P*<0.001).

Levels of Hpr and Hp were determined by ELISA in children from Gabon (aged 1–12 years, 53% male) and Caucasian children (aged 3–4, 47% male) from the United Kingdom. The median concentration of Hpr in Gabon was 0.28 mg/ml which was significantly higher than healthy Caucasian controls (0.049 mg/ml), overall and with age-specific comparisons (Table 1, *P*<0.0001). In Gabon, Hpr levels decreased with increasing age group and Hpr levels were higher in females compared to males (Table 1, *P*<0.001). No differences in Hpr levels with respect to gender were observed in Caucasians (Table 1, *P* = 0.61). Hp levels were higher in Caucasian (median [inter-quartile range] 0.3 mg/ml [0.19–0.48]) compared to Gabonese children (0.13 mg/ml [0.03–0.44]), but this was not statistically significant (*P* = 0.4). In both Caucasian and Gabonese children Hpr was positively correlated with Hp (r_s_ = 0.51, *P*<0.001 and r_s_ = 0.29, *P*<0.001, respectively) (Figure 2).

**Table 1:** Hpr levels in Gabonese and Caucasian children.

	Gabon		Caucasians		
	n	Hpr mg/ml	n	Hpr mg/ml	*P*
Overall	563	0.28 [0.17–0.46]	36	0.049 [0.041–0.069]	<0.001
Age (years)					
1–4	173	0.36 [0.21–0.5]	36	0.049 [0.041–0.069]	<0.001
5–9	318	0.26 [0.17–0.44]			
10–14	61	0.22 [0.15–0.34]			
Sex					
Female	259	0.32 [0.18–0.5]	19	0.054 [0.041–0.08]	<0.001
Male	294	0.25 [0.16–0.4]	17	0.047 [0.041–0.061]	

Values represent median [interquartile range]. P-values represent comparisons between Caucasians and Gabonese children.

### Association between Hpr, Acute Phase Proteins, Parasitaemia and Haptoglobin Polymorphisms in Children from Gabon

Table 2 summarises Hpr, acute phase proteins and parasitological findings of the study children as well as frequencies of haptoglobin polymorphisms. In addition to high Hpr levels, CRP levels were also high in Gabonese children, greater than 80% of whom were defined as having an APR (CRP>10 µg/ml) related to malaria parasitemia. Of note, Hpr levels increased five-fold in Gabonese children with an APR compared with UK children or Gabonese children without an APR. Hp levels were low in this population where 53.5% of children were defined as having hypohaptoglobinemia (Hp<0.18 mg/mL), presumably due to malaria-induced hemolysis [21].

**Table 2:** Acute phase protein levels, parasitaemia and frequency of haptoglobin polymorphisms in Gabonese children.

Variable	All children	
	(n = 553)	
C-reactive protein (µg/mL)	50.3	[13.4–187.3]
C-reactive protein (>10 µg/mL)^1^	444	(80.3)
Haptoglobin (mg/mL)	0.13	[0.03–0.44]
Hypohaptoglobin^2^	296	(53.5)
Albumin (mg/mL)	40.61	(11.5)
Low Albumin^3^	83	(15.1)
*Plasmodium* prevalence	296	(53.6)
Density/µL^4^	800	[286–3314]
Haptoglobin genotype		
1F-1F	92	(16.6)
1F-2	184	(33.3)
1S-1F	91	(16.5)
1S-1S	22	(4.0)
1S-2	85	(15.4)
2-2	79	(14.3)

Continuous data are shown as median value [inter-quartile range] or mean (standard deviation) and categorical data are n (%). Note for C-reactive protein, albumin and plasmodium prevalence n = 550.

1 C-reactive protein >10 µg/mL was the cut-off used to define an acute phase response [17].

2 The cut off value for hypohaptoglobinaemia is <0.18 mg/mL [29].

3 The cut off value for low albumin is <35 mg/mL [30].

4 The frequency of species in this population has been published previously [21].

We wished to identify variables that were associated with Hpr levels at the population level. Variables chosen for investigation were APR proteins (Hp, CRP and albumin), *Plasmodium spp* (prevalence and density), demographics (age and sex) as well as Hp genotype and subtype. Univariate analyses of variables of interest were performed on log (Hpr) levels. Hpr was significantly positively associated with Hp and albumin (*P*≤0.004) but not CRP (*P* = 0.96) (Table 3). Hpr levels decreased with age (*P* = 0.001) and parasitemia (*P* = 0.003). Hpr was also associated with sex and Hp genotype (in the order1–1>2–1>2–2) and Hp subtype (overall *P*≤0.002) (Table 3).

**Table 3:** Univariate assessments of the influence of variables of interest on Hpr levels in Gabonese children.

Variable	*β*	(95% CI)	*P*
Acute Phase Proteins			
Haptoglobin	0.13	(0.09, 0.16)	<0.001
C-reactive protein	0.001	(−0.04, 0.04)	0.96
Albumin	0.015	(0.005, 0.025)	0.004
Haptoglobin genotype			
1–1	0.31	(0.14, 0.49)	0.001
2–1	0.17	(0, 0.34)	0.05
2–2	Reference		
Haptoglobin subtype			
1F-1F	0.41	(0.2, 0.61)	<0.001
1F-1S	0.31	(0.1, 0.51)	0.004
1S-1S	−0.05	(−0.37, 0.27)	0.75
2-1F	0.2	(0.02, 0.38)	0.03
2-1S	0.11	(−0.1,0.32)	0.3
2-2	Reference		
Demographics			
Age	−0.04	(−0.06, −0.02)	<0.001
Males	−0.19	(−0.3, −0.08)	0.001
Females	Reference		
*Plasmodium*			
Positive	−0.16	(−0.28, −0.05)	0.006
Negative	Reference		
Log_10_(Plasmodium+1)	−0.06	(−0.09, −0.02)	0.003

Coefficients are from univariate general linear models investigating the association between variables of interest and log(Haptoglobin-related protein) values.

A multivariate general linear model was then constructed to examine the effect of multiple variables on Hpr levels. The resulting fitted model predicts the median Hpr level for the population controlling for relevant variables. Variables chosen for the final model included age, sex, Hp genotype and Hp concentration. The median Hpr level in the population, after controlling for relevant variables, was 0.27 mg/ml (95% CI 0.25, 0.29). Hpr levels decreased with age (*β* = −0.04, 95% CI 0.06, −0.02, *P*<0.0001) and females had higher Hpr levels (0.29 mg/ml 95% CI 0.27, 0.32) compared to males (0.25 mg/ml 95% CI 0.23, 0.27, *P* = 0.004). Hp levels were positively associated with Hpr levels (*β* = 0.1, 95% CI 0.07, 0.14, *P*<0.0001). Hpr levels also varied according to *Hp^1F^* and *Hp^1S^* alleles (overall *P* = 0.0002). Children with the Hp1-1 genotype, 1S-1S had lower Hpr levels (0.2 mg/ml, 95% CI 0.15, 0.26) than children of 1S-1F (0.28 mg/ml, 95% CI 0.24, 0.33, *P* = 0.09) and 1F-1F genotype (0.29 mg/ml, 95% CI 0.24, 0.36, *P* = 0.018). There was no significant difference in Hpr levels between 2-1F (0.26 mg/ml, 95% CI 0.23, 0.3) and 2-1S genotypes (0.24 mg/ml, 95% CI 0.21, 0.29, *P* = 0.55). Hpr levels in Hp2-2 were 0.21 mg/ml (95% CI 0.17, 0.24) which were significantly lower than 1F-1F, 1F-1S, and 2-1F only.

## Discussion

Data shown represent the first large-scale population study of Hpr levels in humans. Previous laboratory investigations, using a small number of samples, reported lower Hpr levels in the range of 0.02–0.05 mg/ml [15], [27]. This was similar to median levels found in our Caucasian controls (0.049 mg/mL) but significantly lower than those of the Gabonese children (0.28 mg/ml) described in this study. Several explanations can be put forward for these higher levels in Gabonese children. These include host genetics and prevalence of an APR. It is possible that an increase in gene copy number may contribute to higher plasma levels of Hpr in Gabonese compared to Caucasian children. Up to 6 copies of the Hpr gene have been found in the chromosomes of African-Americans, whereas only one copy was found in Caucasian-Americans [32]. The low frequency of increased Hpr gene copy number in African-Americans genotyped (28%) suggests that it is unlikely to account for the relatively high proportion of children with levels of Hpr>0.1 mg/ml (91%) found in this Gabonese study population. The high prevalence of an APR due to malaria infection seems more likely to be the cause of elevated median concentrations, although considerable individual variation in levels was observed, as discussed below.

Hpr levels were found to decrease with age in the Gabonese children. This may be dependent upon intrinsic factors related to the age of the host. It is interesting to note that some acute phase proteins show age-related profiles, e.g. levels of Hp decrease with age, whereas others are unrelated to age, e.g. CRP [33], [34]. Levels of Hpr were higher in females than in males. This finding may be related to metabolism of HDL. It has been shown in a cohort of 10–15 year old males that testosterone decreased levels of HDL-cholesterol and apolipoprotein A-I, whereas estradiol increased levels of HDL-cholesterol [35]. Hpr is found circulating in HDL and thus changes in HDL levels will result in changes in Hpr levels. Furthermore, there is a marked decrease in plasma levels of HDL during infection and inflammation [36].

Univariate analysis showed that Hpr was correlated with the acute phase protein Hp, but not CRP or albumin in Gabonese children. Hp levels are determined by several factors, including IL-6-dependent APR, Hp genotype, parasite density and hemolysis [7], [21]. This correlation between Hpr and Hp would suggest that the production and/or clearance of both these molecules may be under the influence of the same mechanisms. The positive correlations in Hpr and Hp levels in Gabonese children, the majority of whom are undergoing an APR, leads us to suggest that Hpr may also be an APP. Whilst longitudinal analyses within individuals will be required to prove that this is the case, it is plausible that during an APR both Hp and Hpr may be upregulated simultaneously as IL-6 responsive elements (IL-6RE) have been identified in both the Hp and Hpr promoters [19]. Indeed if Hpr is an acute phase protein, it is a moderate one, i.e. a protein showing a 1- to 10-fold increase during an APR as there was a positive correlation with Hp but no direct correlation between Hpr and CRP levels [17]. This is not unexpected; although the CRP gene also contains IL-6RE [37],the relative kinetics of acute phase proteins differ during an APR, IL-6 and IL-1β acting synergistically to induce promotion of CRP production [38]. Thus CRP levels rapidly increase several hundred-fold and fall rapidly whereas Hp levels rise 2-4-fold and persist [17]. Albumin is a negative acute phase protein, i.e. production is reduced in an APR [17].

Hpr levels were inversely correlated with parasitemia. Hp levels also decrease with increasing parasite density due to intravascular hemolysis and subsequent clearance of Hb-Hp complexes [39]. Thus, levels of Hp and Hpr may be correlated due to similar clearance patterns. In support of this possible conclusion, we have also shown that Hpr correlates with Hp in a malaria endemic region of Papua New Guinea, in the absence of an APR (Imrie H, unpublished observations). Whilst Hpr is able to bind Hb, in contrast to Hp-Hb, Hpr-Hb does not promote any high-affinity binding to the scavenger receptor CD163, which clears Hp-Hb complexes from the circulation [40]. Nevertheless, the mechanism of clearance of Hpr-Hb may be related to the clearance of Hp-Hb complexes. It is usual for some plasma Hp (0.9%) to be associated with HDL particles [29] and thus we speculate that when HDL associated Hp binds to Hb, some HDL containing Hpr may be cleared concurrently. Alternatively other scavenger receptors may also exist which bind to both Hp-Hb and Hpr-Hb.

Hpr levels were also associated with Hp genotype; Hp2-2 individuals had significantly lower levels of Hpr compared to Hp1-1 and Hp 2-1 individuals. This may be related to the differential affinity of Hp genotypes for binding Hb and subsequent patterns of clearance. Whilst the affinity of the higher molecular weight polymers for Hb is generally lower than that of Hp1-1, their plasma levels are lower, reflecting the clearance of a greater molecular weight per molecule. Thus levels are in the phenotype order: Hp1-1>Hp2-1>Hp2-2 [9], [41]. Hpr concentration was also related to Hp subtype, with Hp1S-1S having lower levels of Hpr compared to 1F-1F. Functional differences between the Hp sub-phenotypes are unknown and we have reported that subtype (1S or 1F) does not affect plasma concentration of Hp in these children [21].

Our results show that levels of Hpr vary between and within populations from the United Kingdom and Gabon. Since all humans are refractory to infection with *T. b. brucei*, there can be no trypanolytic advantage in having higher levels in endemic areas. The closely-related Hp is a multifunctional protein (e.g. antibody-like properties, immunomodulation, iron metabolism) [42].We speculate that Hpr likewise has more than one role, possibly in innate resistance to other pathogens. Hp levels modulate TLF activity: low Hp levels (due to acute intravascular hemolysis) result in increased TLF activity *in vitro* (10–40 fold), whereas, high levels of Hp suppress both the lytic capacity of TLF *in vitro* and the ability to clear trypanosomes *in vivo* in a mouse model [43]. Thus low Hp levels, as occur in malaria, might also increase other activities of Hpr. We speculate that Hpr may have a role in resistance to malaria. It has been suggested that the binding of Hp-Hb to CD163 elicits IL-6 and IL-10 secretion. It may be that Hpr-Hb complexes also have immunomodulatory effects.

Our population-based observations indicate that a more dynamic view of the relative roles of Hpr and Hpr-Hb complexes needs to be considered in understanding innate immunity to African trypanosomes and possibly other pathogens that cause ahaptoglobinemia, such as the newly discovered *Plasmodium* spp of humans and primates [44].

*In vivo* population data presented here show individual variation in Hpr and Hp levels caused by complex dynamics of Hpr and Hp levels in children living under the burden of malaria infection in African settings with consequences for the ability to control infection.
